# AntiBody Sequence Database

**DOI:** 10.1093/nargab/lqae171

**Published:** 2024-12-18

**Authors:** Simon Malesys, Rachel Torchet, Bertrand Saunier, Nicolas Maillet

**Affiliations:** Institut Pasteur, Université Paris Cité, Bioinformatics and Biostatistics Hub, 28 rue du Dr Roux, F-75015 Paris, France; Institut Pasteur, Université Paris Cité, Bioinformatics and Biostatistics Hub, 28 rue du Dr Roux, F-75015 Paris, France; Unité de Virologie Structurale, Institut Pasteur, Université Paris Cité, CNRS UMR 3569, 28 rue du Dr Roux, 75015 Paris, France; Institut Pasteur, Université Paris Cité, Bioinformatics and Biostatistics Hub, 28 rue du Dr Roux, F-75015 Paris, France

## Abstract

Antibodies play a crucial role in the humoral immune response against health threats, such as viral infections. Although the theoretical number of human immunoglobulins is well over a trillion, the total number of unique antibody protein sequences accessible in databases is much lower than the number found in a single individual. Training AI (Artificial Intelligence) models, for example to assist in developing serodiagnoses or antibody-based therapies, requires building datasets according to strict criteria to include as many standardized antibody sequences as possible. However, the available sequences are scattered across partially redundant databases, making it difficult to compile them into single non-redundant datasets. Here, we introduce ABSD (AntiBody Sequence Database, https://absd.pasteur.cloud), which contains data from major publicly available resources, creating the largest standardized, automatically updated and non-redundant source of public antibody sequences. This user-friendly and open website enables users to generate lists of antibodies based on selected criteria and download the unique sequence pairs of their variable regions.

## Introduction

Humoral immunity is a crucial line of host defense, composed of billions of unique antibodies whose sequences must be adapted to specific pathogens (1). Sequences of antibodies are widely studied as a key element of the immune response (2). In 2024, in humans, only a few thousands protein sequences are publicly accessible in databases.

With the rapidly increasing usage of artificial intelligence (AI), the need for standardized datasets has never been so high. Regarding antibodies, this means obtaining the sequences of as many unique pairs of antibody variable regions as possible, according to specific keywords in the sequence metadata. For example, building datasets that contain unique antibodies linked to specific diseases or species, or only monoclonal antibodies with an INN (3) ID (i.e., not immunoglobulins found only in B cells), etc.

However, retrieving available antibody sequences to build proper datasets is by no means straightforward. Antibody sequences are spread across several databases, making it difficult to compile the sequences of interest into unique datasets. Additionally, there is a significant sequence redundancy within and between databases. Several databases are based on the PDB (4), resulting in data redundancy, and the PDB itself contains several identical antibody sequences registered with different PDB IDs (e.g., PDB IDs 7WVM and 8GY5). Poor metadata quality, for example containing errors or omitting crucial information, raises additional concerns.

Here, we present ABSD (AntiBody Sequence Database, https://absd.pasteur.cloud), which addresses the downsides mentioned above. Protein sequences from publicly available resources, including Kabat (5), IMGT (6), the PDB, UniProt (7), GenBank (8), The Observed Antibody Space database (OAS) (9) and others, are used to build the largest standardized and non-redundant public resource of antibody variable regions (paired light and heavy chain sequences). The database presented here is automatically updated and was designed to be easily upgraded, making it simple for developers to add new species or introduce new data sources. To create lists of desired antibodies (according to different criteria), this user-friendly website enables downloading selected data in just a few seconds.

## Materials and methods

Recovering and formatting antibodies from different sources into a single collection is not straightforward. This is performed in two distinct steps. The first step is source-dependent and consists in extracting relevant information from each database. The second step involves taking produced results from the first step and merging all information into a single, non-redundant collection of antibodies. Both steps are described in more detail below. Then, the database and website implementation is presented.

### Extracting data

From each database, a file containing as many antibodies as possible is extracted. The data extraction method varies depending on the database (refer to Supplementary Data for more information). Each file is then processed independently using an ad hoc script. For example, the script for the PDB and *Homo sapiens* species selects only sequences with headers containing keywords ‘homo’ or ‘sapiens’ and at least one of the following: ‘light’, ‘kappa’, ‘lambda’ (denoted as *‘light’ keywords* in the rest of the article), ‘heavy’, ‘alpha’, ‘gamma’, ‘delta’, ‘epsilon’ or ‘mu’ (denoted as *‘heavy’ keywords* in the rest of the article). Sequences with headers containing irrelevant keywords (e.g., ‘thrombin’, ‘antigen’, ‘subcomponent’, etc.) are discarded. Selected sequences are grouped by IDs. Occasionally, a certain level of redundancy can be found between sequences grouped together. For example, the sequence with PDB ID 5XAJ_4 is fully included in 5XAJ_5. In such scenarios, the smallest sequence is discarded. After these extraction and cleansing steps, antibodies need to be paired two by two.

#### Pairing IgGs

To properly pair light and heavy chain sequences within an ID group, metadata in headers is used by the script; for example, in the group 8AON, the sequence 8AON_3|Light chain|Homo sapiens (9606) is paired with 8AON_2|Heavy chain|Homo sapiens (9606). Nevertheless, some headers proved more difficult, and four strategies were developed to maximize the number of paired sequences.

The first strategy pairs two sequences when the header of one is fully included in another one, modulo ‘heavy’ and ‘light’ keywords. With this first strategy, sequences sharing identical headers (or with slight differences) are paired. For example, 2HFG_1|CB3s Fab light chain (kappa)|Homo sapiens (9606) is paired with 2HFG_2|CB3s Fab heavy chain|Homo sapiens (9606). This strategy is the most successful one (except for OAS data that will be described later), pairing 80,034 antibodies (human: 59 038, mouse: 20 996) in total.

The second strategy is applied to remaining groups of only two sequences. If one header has a ‘heavy’ keyword and the other one a ‘light’ keywords, corresponding sequences are paired. For example, this strategy pairs 5DRX_2|CLL240 BCR light chain|Homo sapiens (9606) with 5DRX_1|CLL240 heavy chain (VH and CH1 domains)|Homo sapiens (9606). In total, this strategy pairs 2639 antibodies (human: 2421, mouse: 218).

The third strategy is applied to remaining groups. It uses previously paired antibodies in ABSD (from any sources). For each group, if two sequences are equal, are included in or include two previously paired sequences, they are paired. This method allows bypassing some errors in the headers. For example, this strategy successfully pairs the light chain 7URS_3|COV11 Fab HEAVY CHAIN|Homo sapiens (9606) with 7URS_2|COV11 Fab HEAVY CHAIN|Homo sapiens (9606), using the previously paired antibody 7S4S from IMGT and the PDB. In total, this strategy pairs 297 antibodies (human: 189, mouse: 108).

The last strategy is used in the most challenging cases, where all other approaches have proven unsuccessful. The script then proposes manual pairing of sequences, one group at a time. For example, for the group 1CL7, the automatic pairing of 1CL7_1|IGG1 ANTIBODY (light chain), 1CL7_2|IGG1 ANTIBODY (variable heavy chain) and 1CL7_3|IGG1 ANTIBODY (constant heavy chain) evades all previous methods but can easily be done by a human. In total, this strategy pairs 357 antibodies (human: 212, mouse: 145).

This script then cleanses and standardizes the data as follows:

#### Standardizing sequences

Until this stage, sequences were paired solely based on metadata, which sometimes contain errors. A list of known starting patterns of antibody variable regions was extracted from IMGT and manually enriched with recurring patterns. Using this list, the script examines each sequence and corrects pairs where a sequence header was mistakenly labeled as ‘light’ instead of ‘heavy,’ and vice versa. For instance, both headers of 5TPP are incorrectly annotated in the PDB. In total, 389 antibodies were corrected (human: 210, mouse: 179).

Using the list of expected starting patterns and a similar list of expected ending patterns, the script truncates sequences to retain only the largest portions, starting and ending with known patterns. At the same time, unrecognized sequences are discarded. This step has a twofold purpose: it standardizes the data to retain only variable region, while removing some sequences wrongly identified as antibodies. In total, 1377 pairs are discarded (human: 642, mouse: 735).

Finally, the script discards pairs where at least one of the sequences is <80 amino acids or >150 amino acids. In total, 880 antibodies are discarded (human: 567, mouse: 313).

In total, 81 067 antibodies (human: 60 648, mouse: 20 419) were extracted from the different databases. The results were compiled into two FASTA files per database, one containing all the ‘light’ chain sequences and the other all the ‘heavy’ chains, in the same order to preserve the pairing information.

Although PLAbDab used several strategies to pair heavy and light chain Fv sequences (see *Creating paired antibody sequences from unpaired data* (10)), the strategies followed by ABSD yielded similar result: 33 759 pairs of antibodies (human: 26 150, mouse: 7609) were extracted. A total of 1833 antibodies (human: 1006, mouse: 827) were subsequently discarded, either because they lacked a start/end pattern or were too small. A total of 31 926 antibodies (human: 25 144, mouse: 6782) were extracted from PLAbDab.

In addition, sequences from OAS were all paired using the second strategy (groups of only two sequences with one ‘heavy’ keyword and one ‘light’ keywords). A total of 1 982 578 antibodies (human: 1 954 071, mouse: 28 507) were paired. A total of 4076 antibodies (human: 4044, mouse: 32) were discarded either by lacking a start/end pattern or being too small. In total, 1 978 502 antibodies (human: 1 950 027, mouse: 28 475) were extracted from the OAS database.

### Merging data

When this process is completed for several datasets, data must be merged together. Another script removes duplicate sequence pairs. This includes identical sequences as well as sequences that are fully contained within each other. For example, if the light sequence of an A pair is included in the light sequence of a B pair, and both heavy sequences are identical or included one in the other, only the longest light and heavy sequences are retained, forming the antibody. At the same time, original headers are merged, preserving metadata and tracing data sources. For instance, at the end of the header of the light A sequence, a source tag from where A was extracted is added (e.g. ‘;PDB’), followed by a separator in the form of three pipes (‘|||’), then the header of the light B sequence is added, followed by its own source tag (e.g., ‘;IMGT’). This becomes the final header of the light sequence for this merged antibody. This merging step eliminates redundancy, and the number of antibodies decrease from 2 059 569 (human: 2 010 675, mouse: 48 894) to 1 849 280 (human: 1 825 254, mouse: 24 026).

Another difficulty arises at this step. Among the 1 825 254 human antibodies extracted (and 24 026 for mouse), we can only identify 2 551 639 unique sequences (and 32 446) instead of the expected 3 650 508 (and 48 052, two sequences per antibody). The missing sequences are due to differences in databases. Sometimes, a light (or heavy) chain is paired with several different heavy (or light) chains. For example, PDB IDs 4RRP, 5UEA and 6AZ2 have identical light chains and almost identical heavy chains: the only difference lies in the end of the heavy chain, which is vtvSS for 4RRP, vtvVS for 5UEA and vtvF for 6AZ2. This particular case is easily resolved by taking the ‘longest common substring’ between all lights (or heavy) chains to form a merged antibody and then ensure that this antibody passes all the standardizing procedures described in *Standardizing sequences*.

When differences are not located at the beginning or ending of sequences, the ‘longest common substring’ strategy does not work. For instance, PDB IDs 12E8 and 3JBQ share the same light chain but have a small divergence in the heavy chain. In the middle of these sequences, 12E8 has the sub-sequence WIDPEI, while 3JBQ has only WIDEI, missing a Proline. In such situations, it is not possible to make an automatic choice; these immunoglobulins are called ambiguous and were gathered in clusters that are not included in ABSD. A group of antibodies is then defined as an ‘ambiguous’ cluster of antibodies when they share, two to two, a common chain sequence (this sequence is not necessarily the same for the whole cluster).

Even more complex situations exist where a light chain is paired with two different heavy chains (PDB IDs: 4YGV and 4YHO), and these heavy chains are also paired with different light chains in other pairs (PDB IDs: 4YGV and 4YHL). These situations resulted in the formation of clusters of ambiguous immunoglobulins that were not included in ABSD. Of note, out of the 1 825 254 human antibody sequences analyzed, only 768 584 were found to be non-redundant and unambiguous. This discrepancy could be a characteristic of the OAS database, which uses nucleotide sequences translated into protein sequences. Most human sequences included in OAS originated from 12 studies that each involved only a few subjects. Therefore, many sequences originated from just a handful of individuals. This increases the probability to sequence BCRs from related germlines, which will yield various combinations of identical heavy/light chains (ambiguous). But it is unlikely that all these combinations would be selected to produce mature antibody.

A final source of redundancy lay in identical sequences shared by different species. For example, 5CJO (*Mus musculus*) and 4M1C (*Homo sapiens*) have identical light chains and almost identical heavy chains (with the addition of two serines at the end of the human heavy chain). It is annotated as a ‘FAB Heavy chain with engineered elbow’ in the mouse dataset. Other duplicates contain, among others, some monoclonal antibodies, humanized antibodies, mouse/human chimera, etc. This final source of redundancy involves 581 antibody sequences; these sequences and their corresponding antibodies have been removed from ABSD.

In total, ABSD contains 774 662 antibodies (1 549 324 sequences). There are 768 285 non-ambiguous, unique human antibodies (1 536 570 sequences), plus 73 308 that are difficult to resolve and are organized in 51 721 clusters (2 551 639 unique sequences); additionally, there are 6377 non-ambiguous, unique mouse antibodies (12 754 sequences), plus 2359 that are difficult to resolve and are organized in 1351 clusters (32 446 unique sequences).

### V gene segments

To determine the origin of the V gene segments in ABSD, reference databases for igBLAST were constructed by downloading data from the IMGT website (https://www.imgt.org/vquest/refseqh.html). Specifically, two databases were created per species (human and mouse): one for the heavy chains (comprises for Immunoglobulin Heavy V gene segments (IGHV)) and one for the light chains (comprises for Immunoglobulin Kappa light V gene (IGKV) and Immunoglobulin Lambda light V gene (IGLV) segments). Subsequently, each sequence was analyzed using igBLAST, and the V gene segment corresponding to the best-hit was appended to the sequence metadata.

### Organizing the database

Sequences are stored in a MongoDB database, which saves entries in JSON format. Each entry corresponds to an antibody, comprising both light and heavy chains, along with its associated metadata, such as species, the list of source databases and FASTA headers.

Due to the absence of a reliable unique identifier for each antibody, a custom one is created for every entry. It consists of a SHA-256 hash computed from a string made by concatenating the species and the light and heavy chain sequences, ensuring uniqueness for every antibody, even if more are to be added in the future.

## Results and discussion

The ABSD website (https://absd.pasteur.cloud) is publicly available and guarantees the uniqueness of each pair of antibody chain sequences within its database. In this article, the representativeness of the current dataset was evaluated by comparing the clustering of its VH (heavy chains’ V) gene segments to those of published human antibody repertoires. The article also discusses the issues of redundancy in original data sources, as well as those of missing data. Furthermore, it explains how ABSD is automatically updated and can be easily upgraded with new resources or expanded to additional species, and highlight the ongoing difficulty of extracting data from some databases (not yet fully addressed).

### Representativeness of **ABSD** antibody dataset

To assess the representativeness of antibody sequences compiled in ABSD, the frequency with which each of the seven clusters of V gene segments (VH) encoded their heavy chain variable regions, was compared to that of the *Preprocessed data* from Horns *et al.* (11). Note that, although V is the longest gene segment, the variable regions result from random recombination of sets of three (two, for light chains) distinct gene segments named V (D) and J. In the latter study, B cells were sampled at different time-points from the bloodstream of five individuals to study their BCR repertoire (B-cell receptors are immunoglobulins expressed on their surface). In total, the nucleotide sequences of 1 847 213 BCRs were identified, including various isotypes, which correspond to their constant regions (mainly IgG, IgA and IgM). Using igBLAST (12), the portions of heavy chain protein sequences encoded by V gene segments (IGHV) from this dataset were compared to those of the human IGHV gene segments available in IMGT (https://www.imgt.org/vquest/refseqh.html).

**Figure 1.**
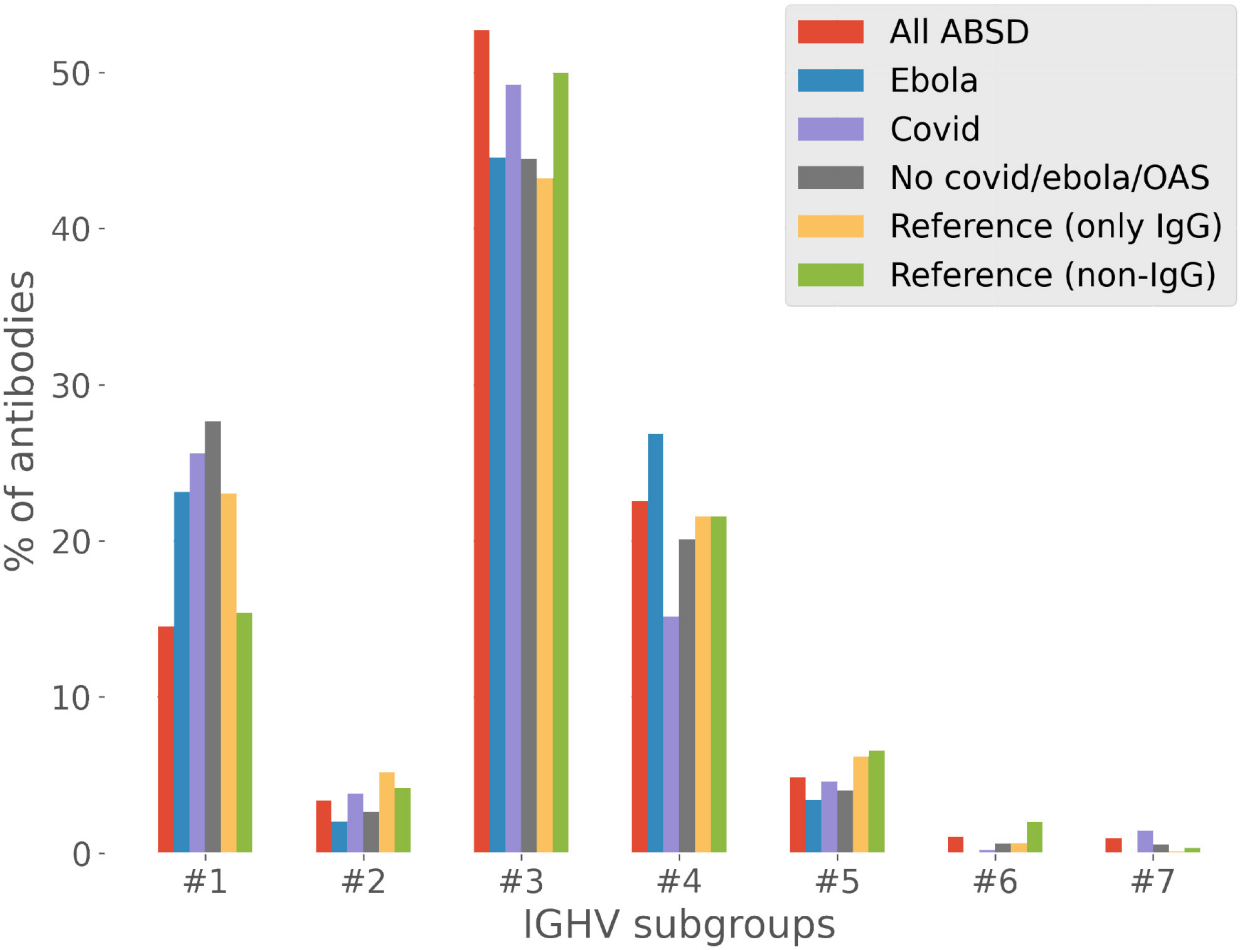
IGHV percentages in ABSD’s human antibody sequences and those from known repertoires. The sequences of heavy chain variable regions from the ABSD and reference datasets were sorted in seven IGHV clusters. The percentages of each of them was calculated for the whole ABSD antibody dataset (‘*All ABSD*’), or subsets as displayed on the graph, as well as for the reference dataset (‘*Reference*’); ‘*Ebola*’ and ‘*Covid*’ refer to subsets of antibody sequences identified in patients infected by either virus.

About two-thirds of each heavy chain variable region is encoded by an IGHV belonging to one of seven clusters of gene segments, classified according to their phylogeny. The variable sequence of each heavy chain is then catalogued into one of the seven IGVH clusters identified, based on sequence similarities, using the best hit of the IgBLAST result. This analysis was performed separately for the IgG sequences of the dataset on one hand (‘Reference (only IgG)’ in Figure 1) and other Ig isotypes on the other hand (‘Reference (non-IgG)’ in Figure 1). Then, four distinct analyses were performed using the antibody dataset from ABSD. The first analysis (‘All ABSD’ in Figure 1) involved all human heavy chain sequences compiled in ABSD (768 285 sequences), the second analysis (‘Ebola’ in Figure 1) only included antibodies coming from one Ebola study (13) (294 sequences), the third analysis (‘Covid’ in Figure 1) only included antibodies coming from the CoV-AbDab (14) database (8369 sequences), and the last analysis (‘No covid/ebola/OAS’ in Figure 1) involved antibodies that came neither from the two previous analysis nor from OAS database (17 352 sequences).

The Ebola group was selected for three reasons: it is disease-related, small in size (comprised of only 294 antibodies), and contains only antibodies originating from GenBank. These parameters could have introduced biases, potentially showing antibodies preferentially coming from one subgroup. Similarly, antibodies coming from the CoV-AbDab database are disease-related and also represent an important group of antibodies coming from a single database. Again, these features could have introduced biases. However, the results reveal that for all four samplings, the proportion of antibodies coming from each subgroup is consistent with what was anticipated. It is still unclear why the inclusion of data from the OAS database led to proportions of each class of V gene segments similar to the values observed with non-IgG sequences of the reference dataset. The most straightforward explanation is that these sequences were mostly coming from memory B cells that have not yet undergone IgM class switch, as opposed to most B cells already secreting antibodies.

### Non-ambiguous and unique antibodies

To the best of our knowledge, ABSD is the only public database that exclusively contains non-redundant sequences, thereby providing unique antibody sequences. As shown in Table 1, all databases used in this study originally contained a certain level of redundancy or ambiguous antibody sequences. For instance, out of the 3632 human antibodies extracted from the PDB, less than half (1494) were actually non-ambiguous, unique antibody sequences.

**Table 1.** Number of antibodies retrieved per database (up-to-date on 21 September 2024)

	Total extracted		With unique sequences	
Database	Human	Mouse	Human	Mouse
AbDb (15)	1237	1170	491	372
AbPDB(15)	861	989	333	324
Cov-AbDab (14)	10 041	297	8304	151
CoV-AbDab-PDB (14)	729	85	295	36
EBOLA (8,13)	321	0	294	0
IMGT (6)	7904	4344	1585	594
Kabat (5)	465	892	310	513
OAS (9)	1 950 027	28 475	744 648	2812
PDB (4)	3632	1964	1494	639
PLAbDab (10)	25 144	6782	14 351	2939
SACS (15)	3501	1935	1456	630
SAbDab (16)	3578	1961	1480	635
TheraSAbDab (17)	1170	0	770	0
UniProt (7)	2065	0	1875	0
Total	2 010 675	48 894	777 686	9645
Non-ambiguous			768 285	6377

### Redundancy in sources

**Figure 2.**
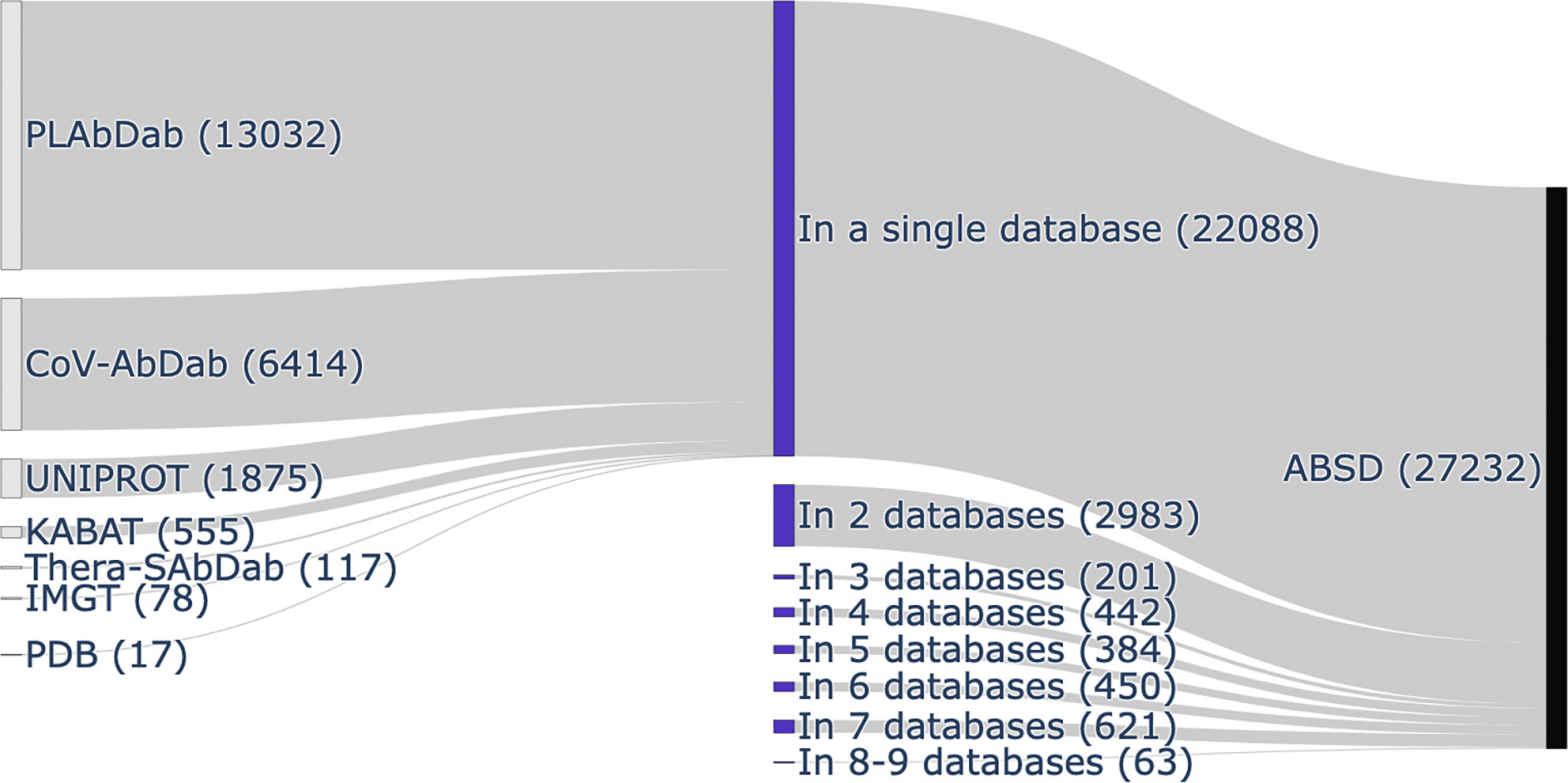
Proportions of ABSD’s antibody sequences in original databases. The right part represents the proportions of the whole ABSD dataset extracted from shown databases (for the sake of clarity, data from the OAS database were excluded; for a comprehensive representation, see Supplementary Figure S1). The middle part represents the proportions of antibody sequences present in only one (top) or multiple (bottom) databases: overall, the latter represent ∼20% of ABSD’s dataset; the names of the databases containing the remaining 80% of unique sequences are displayed at the left.

Although each sequence is unique, many antibody sequences are present in multiple sources used by ABSD. Figure 2 illustrates that while most antibodies come from a single database, approximately one-fifth of them (5144/27 232) are found in multiple sources. Most antibody sequences from PLAbDab (13 032/17 290), CoV-AbDab (6414/8455), UniProt (1875/1875) or from Kabat (555/823) are exclusive to these databases; for the sake of clarity, data from OAS were not displayed in the representation (for an exhaustive representation, see Supplementary Figure S1). In contrast, antibody sequences in the PDB are sometimes redundant and found also in different databases; however, most of the latter actually originated from the PDB. Similarly, data from Thera-SAbDab are almost entirely included in the IMGT dataset (653/770). By maintaining a record of the source for all antibody sequences when information was merged in ABSD, users can directly access all the databases from which an entry was extracted.

### Updating and improving **ABSD**

ABSD comprises data compiled from several sources, including major databases like the PDB, Kabat, OAS or IMGT. Due to its fully automated extraction (cf. *Extracting data* in Ms&Ms) and merging (see *Merging data*) steps, ABSD is automatically updated once a month and can be easily upgraded. For instance, incorporating a new database only requires developing a simple parser to extract the data into a specific format. Once accomplished, the new data are automatically standardized and integrated into the existing database. Similarly, adding antibody sequences from additional species to ABSD is a straightforward process.

Note that some databases have proven to be more challenging to extract data from. For instance, all antibody sequences collected from GenBank and UniProt were limited to either the light or heavy chain, but not both. Since pairing information is often missing in these cases, it was impossible to reconstruct the full antibody sequence for most of them.

## Conclusion

While several databases enable collecting antibody protein sequences, consolidating data from various sources has hitherto remained a challenge. Here, we introduce ABSD, which addresses this issue while ensuring that each antibody sequence stored in the database is unique and standardized.

With our methodology, we likely missed several immunoglobulin sequences, especially when essential information in the metadata (such as chain type, species, etc.) was already missing, misleading or contained errors.

An additional potential bias to consider is that ABSD contains >770 000 antibody sequences, while trillions of them may circulate in the human population. This limitation is unlikely to be resolved anytime soon, but diversity might matter more than shear number. At least regarding IGHV regions, our methodology does not seem to have introduced a strong bias in the selection of antibody sequences towards specific gene clusters, compared to a classic human repertoire. In a near future, modelling light and heavy chain CDR3 regions may help partially overcoming the lack of sampling.

These qualities of uniqueness and relative representativeness of the human repertoire offered by ABSD may be critical for certain applications. Specifically, when training deep learning models, it is essential to use input data that is as unbiased as possible. Therefore, ABSD facilitates this critical step by providing unique antibody sequences with realistic proportions that reflect the human repertoire.

Finally, ABSD is a dynamic and adaptative database, designed for automatic update and easy upgrade. Given the rapidly growing interest in antibodies in recent years (1,2), it is crucial to provide datasets with up-to-date, unique, and non-redundant antibody sequences.

## Supplementary Material

lqae171_Supplemental_File

## Data Availability

ABSD is licensed under the GNU GPLv3 and is publicly accessible at https://absd.pasteur.cloud. It follows the software development standards, featuring continuous integration on Gitlab and comprehensive documentation (https://gitlab.pasteur.fr/hub/absd). The parsers, scripts for data extraction and merging, and tests are openly available at https://gitlab.pasteur.fr/hub/absd/-/tree/master/parsers, allowing anyone to contribute and add new sources. Further implementation details can be found in the project repository at https://gitlab.pasteur.fr/hub/absd. The code has been added to the Software Heritage initiative, for long-term preservation, and is accessible at https://archive.softwareheritage.org/swh:1:dir:c1aaf7ff6fb6deb92ad216b7cbc9d6381e575689.

## Reproducibility

All scripts acquired and generated data in date of the 21 September 2024 are available for reproducibility purpose at https://doi.org/10.57745/DDLHWU. Cleaned and formatted data used for the database construction in date of the 21 September 2024 are also available in data folder (https://gitlab.pasteur.fr/hub/absd/-/blob/master/data/2024-09-21.tar.gz).
