# Supplementary material for: AntiBody Sequence Database
Source: NAR Genom Bioinform. 2024 Dec 18;6(4):lqae171. doi: 10.1093/nargab/lqae171 (PMC11655285; doi:10.1093/nargab/lqae171)
Supplement: lqae171_Supplemental_File [file lqae171_supplemental_file.pdf]

Supplementary figure 1 - All data from **ABSD**

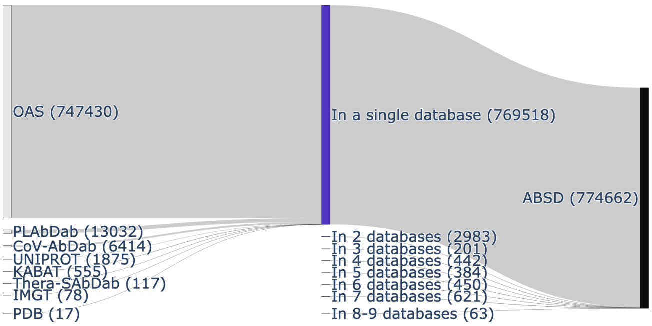

Fig. 1: Proportions of **ABSD**'s antibody sequences in original databases. The right part represents the proportions of the whole **ABSD** dataset, extracted from given databases. The middle part represents the proportions of antibody sequences present in only one (top) or multiple (bottom) databases.

Supplementary data

Data acquisition for ABDB

Data from `http://www.abybank.org/abdb/` (**Download Dataset** tab) can be retrieve by downloading the **Complete Dataset** of **Redundant Antibody List** (`http://www.abybank.org/abdb/Data/Redundant_files/Redundant_LH_Combined_Martin.txt`).

From this file, all PDB IDs (the 4 characters preceding the _) were used to query the PDB (`https://www.rcsb.org/downloads/fasta`) and all results were merged into a single FASTA file.

Data acquisition for AbPDB

From `http://www.abybank.org/abpdbseq/` (file **abpdbseq_latest.faa**), all PDB IDs (the 4 characters preceding the _) were used to query the PDB (`https://www.rcsb.org/downloads/fasta`) and all results were merged into a single FASTA file.

Data acquisition for CoV-AbDab

From `https://opig.stats.ox.ac.uk/webapps/covabdab/`, a csv file can be accessed from **Downloads** tab, **Database (CSV)**.

Data acquisition for CoV-AbDab-PDB

From `https://opig.stats.ox.ac.uk/webapps/covabdab/`, a folder with PDB sequences can be accessed from **Downloads** tab, **PDB Structures (.tar.gz)**. From this file, all PDB IDs (the 4 characters preceding the _) were used to query the PDB (`https://www.rcsb.org/downloads/fasta`) and all results were merged into a single FASTA file.

Data acquisition for EBOLA

IDs were obtained from Table S7 of Supplementary Materials of *Isolation of potent neutralizing antibodies from a survivor of the 2014 Ebola virus outbreak*. Data were then retrieved from GeneBank (`https://www.ncbi.nlm.nih.gov/genbank/`).

Data acquisition for IMGT-INN

From the folder obtained at `https://www.imgt.org/download/3Dstructure-DB/IMGT3DFlatFiles.tgz`, the INN (ungzipped) files are retrieved. The PDB (ungzipped) files were used to retrieve IDs (the 4 characters preceding the _) to then query the PDB (`https://www.rcsb.org/downloads/fasta`) and all results were merged into a single FASTA file.

Data acquisition for IMGT

Data are obtained from `https://www.imgt.org/3Dstructure-DB/` by setting **Display results** to *Domains and sequence alignment* and **IMGT complex type** (in **IDENTIFICATION**, in **Search using IMGT-ONTOLOGY concepts**) to *IG/Ag*. The result page is copied into a text file.

Data acquisition for IMGT2

Data are obtained from a dump of `https://www.imgt.org/3Dstructure-DB/` by setting **Display results** to *Domains and sequence alignment* and **IMGT receptor type** (in **IDENTIFICATION**, in **Search using IMGT-ONTOLOGY concepts**) to *IG*. The result page is copied into a text file.

Data acquisition for KABAT

The file was obtained from `http://www.abybank.org/kabat/`.

Data acquisition for OAS

Files are obtained from `https://opig.stats.ox.ac.uk/webapps/oas/oas_paired/`, when clicking "Search" without using any attributes, then clicking on here link.

Data acquisition for PLAbDab

Data are obtained from `https://opig.stats.ox.ac.uk/webapps/plabdab/static/downloads/paired_sequences.csv.gz`.

Data acquisition for PDB

All IDs were taken from the PDB advanced search page (`https://www.rcsb.org/search/advanced`) by searching **Macromolecule Name**, **has any of words**, *light heavy*. All PDB IDs (the 4 characters preceding the _)) were used to query the PDB (`https://www.rcsb.org/downloads/fasta`) and all results were merged into a single FASTA file.

Data acquisition for SAbDab

From `https://opig.stats.ox.ac.uk/webapps/sabdab-sabpred/sabdab/search/?all=true#downloads`, the link **Download the summary file** (`https://opig.stats.ox.ac.uk/webapps/sabdab-sabpred/sabdab/summary/all/`) was used. From this file, all PDB IDs were used to query the PDB (`https://www.rcsb.org/downloads/fasta`) and all results were merged into a single FASTA file.

Data acquisition for SACS

From `http://www.abybank.org/sacs/` (**Download Chain List**), the **antibodies.txt** file was retrieved. From it, all PDB IDs (the 4 characters preceding the _) were used to query the PDB (`https://www.rcsb.org/downloads/fasta`) and all results were merged into a single FASTA file.

Data acquisition for TheraSAbDab

From Thera-SAbDab page `https://opig.stats.ox.ac.uk/webapps/sabdab-sabpred/therasabdab/search/?all=true`, the csv file was downloaded (`https://opig.stats.ox.ac.uk/webapps/sabdab-sabpred/static/downloads/TheraSAbDab_SeqStruc_OnlineDownload.csv`).

Data acquisition for UNIPROT

A fasta file was generated from `https://www.uniprot.org/uniprotkb?query=Immunoglobulin%2C%20Antibody%2C%20IG`.
